# A functional genomic model for predicting prognosis in idiopathic pulmonary fibrosis

**DOI:** 10.1186/s12890-015-0142-8

**Published:** 2015-11-21

**Authors:** Yong Huang, Shwu-Fan Ma, Rekha Vij, Justin M. Oldham, Jose Herazo-Maya, Steven M. Broderick, Mary E. Strek, Steven R. White, D. Kyle Hogarth, Nathan K. Sandbo, Yves A. Lussier, Kevin F. Gibson, Naftali Kaminski, Joe G. N. Garcia, Imre Noth

**Affiliations:** Section of Pulmonary & Critical Care Medicine, University of Chicago, 5841 S. Maryland Avenue, Chicago, IL 60637-6076 USA; Pulmonary, Critical Care and Sleep Medicine, Yale University, New Haven, CT USA; Institute for Genomics and Systems Biology, University of Chicago, Chicago, IL USA; Department of Medicine, Bio5 Institute, UA Cancer Center, University of Arizona, Tucson, AZ USA; Division of Pulmonary, Allergy and Critical Care Medicine, University of Pittsburgh, Pittsburgh, PA USA; Arizona Respiratory Center and Department of Medicine, The University of Arizona, Tucson, AZ USA

**Keywords:** Idiopathic pulmonary fibrosis (IPF), Peripheral blood mononuclear cells (PBMCs), Gene expression profiling, Functional genomic model, Prognosis prediction

## Abstract

**Background:**

The course of disease for patients with idiopathic pulmonary fibrosis (IPF) is highly heterogeneous. Prognostic models rely on demographic and clinical characteristics and are not reproducible. Integrating data from genomic analyses may identify novel prognostic models and provide mechanistic insights into IPF.

**Methods:**

Total RNA of peripheral blood mononuclear cells was subjected to microarray profiling in a training (45 IPF individuals) and two independent validation cohorts (21 IPF/10 controls, and 75 IPF individuals, respectively). To identify a gene set predictive of IPF prognosis, we incorporated genomic, clinical, and outcome data from the training cohort. Predictor genes were selected if all the following criteria were met: 1) Present in a gene co-expression module from Weighted Gene Co-expression Network Analysis (WGCNA) that correlated with pulmonary function (*p* < 0.05); 2) Differentially expressed between observed “good” vs. “poor” prognosis with fold change (FC) >1.5 and false discovery rate (FDR) < 2 %; and 3) Predictive of mortality (*p* < 0.05) in univariate Cox regression analysis. “Survival risk group prediction” was adopted to construct a functional genomic model that used the IPF prognostic predictor gene set to derive a prognostic index (PI) for each patient into either high or low risk for survival outcomes. Prediction accuracy was assessed with a repeated 10-fold cross-validation algorithm and independently assessed in two validation cohorts through multivariate Cox regression survival analysis.

**Results:**

A set of 118 IPF prognostic predictor genes was used to derive the functional genomic model and PI. In the training cohort, high-risk IPF patients predicted by PI had significantly shorter survival compared to those labeled as low-risk patients (log rank *p* < 0.001). The prediction accuracy was further validated in two independent cohorts (log rank *p* < 0.001 and 0.002). Functional pathway analysis revealed that the canonical pathways enriched with the IPF prognostic predictor gene set were involved in T-cell biology, including iCOS, T-cell receptor, and CD28 signaling.

**Conclusions:**

Using supervised and unsupervised analyses, we identified a set of IPF prognostic predictor genes and derived a functional genomic model that predicted high and low-risk IPF patients with high accuracy. This genomic model may complement current prognostic tools to deliver more personalized care for IPF patients.

**Electronic supplementary material:**

The online version of this article (doi:10.1186/s12890-015-0142-8) contains supplementary material, which is available to authorized users.

## Background

Idiopathic pulmonary fibrosis (IPF) is a fibrotic interstitial lung disease characterized by irreversible scarring of the lung parenchyma that predominantly affects older adults. While older retrospective studies suggested median survival was 2–3 years [1–3], IPF has a highly heterogeneous disease course, making prognostication difficult [4, 5]. While lung transplantation remains the sole intervention to prolong survival in patients with IPF [6], organ scarcity, and ineligibility secondary to comorbid health conditions, make this available to only a few. Pirfenidone [7] and nintedanib [8] have emerged as promising therapies that slow disease progression. Several other medications are currently under investigation. Without the ability to predict disease course, it is difficult to identify which IPF patients are most likely to benefit from these new therapies or from lung transplantation.

Many clinical parameters, including race, gender, age, radiographic and/or histopathologic patterns, and pulmonary function tests have been linked to prognosis in patients with IPF [9, 10]. Lung tissue-based molecular genomic signatures [11, 12] have also been used to predict IPF progression; however, given the resources needed to perform lung biopsy and risk associated with the procedure, the applicability of such genomic signatures is limited. Peripheral blood mononuclear cells (PBMC), comprised of circulating monocytes, T-cells, B-cells, and natural killer cells, have been successfully used as an alternative for exploratory transcriptional profiling studies [13–15]. Advantages of using PBMC over lung biopsy specimens to delineate molecular mechanisms of IPF include easier access, larger quantities, and the ability to dynamically assess disease status through longitudinal sample collection.

Using PBMC gene expression profiling, our group previously identified a genomic signature consisting of 52 genes that predicted survival in patients with IPF [16]. While this investigation drew attention to the potential role of T cell signaling in IPF progression, the contribution of other genes identified in the study were not addressed. Furthermore, the gene set identified from our previous study did not provide a weighted score for the gene expression pattern, which has the potential to be useful in practical application. We therefore aim to construct a functional genomic model to better predict prognosis of IPF patients. To do so, we compiled a set of IPF prognostic predictor genes from previously reported microarray data in the training cohort (accession number GSE28221) [17, 18]. First, we coupled PBMC gene expression profiling to IPF clinical traits using an unbiased “Weighted Gene Co-expression Network Analysis (WGCNA)” approach which is useful for describing the pairwise correlated expression among gene transcripts with co-regulation implications [19–21] and to restrict the search space of genes to those genes in modules associated with pulmonary function. Second, we performed a supervised “Significance Analysis of Microarray (SAM)” approach to identify differentially expressed genes between observed “good” vs. “poor” prognosis IPF patients. Third, we identified genes based on their association with survivorship. The IPF prognostic predictor gene set satisfying all aforementioned three functional genomic criteria was used to construct a genomic prediction model and derived a prognostic index (PI) score for each patient in the training cohort. We then assessed the prognostic prediction specificity in the training cohort and further validated it in two independent cohorts. This work produced a functional genomic model with a mechanism-anchored IPF prognostication score for each patient, which may better identify those most likely to benefit from IPF-specific therapy and provide a tool for personalized IPF management.

## Methods

### Study populations

Study populations were collected, as previously described, from the University of Chicago Medical Center (UCMC ) and University of Pittsburg Medical Center (UPMC) [16]. The training cohort consisted of 45 individuals with IPF collected from November 2007 to July 2009 at UCMC. The University of Chicago validation cohort (UCV) consisted of 21 individuals with IPF along with 10 healthy control subjects without lung disease collected from February 2007 to October 2007. The University of Pittsburg validation cohort (UPV) consisted of 75 individuals with IPF collected from March 2001 to September 2010. While the site source of the samples overlapped between cohorts, all samples were independent of each other. All patients with IPF met American Thoracic Society/European Respiratory Society (ATS/ERS) diagnosis criteria [2]. The local Institutional Review Boards at the University of Chicago and University of Pittsburg Medical Center approved the study and informed consent was provided by all study subjects.

Demographic information, clinical characteristics, and pulmonary function tests were collected from all patients with IPF. Spirometry testing, including forced vital capacity percent predicted (FVC% predicted), diffusion capacity for carbon monoxide percent predicted (D_L_CO % predicted) as well as lung volumes by plethysmography were obtained per ATS guidelines [19–21]. The composite physiologic index (CPI) was calculated as described by Wells et al. [22]. Survivorship was obtained from medical records, telephone interviews, and the social security death index database. The prognosis of IPF subjects was dichotomously categorized as good or poor based on observed survival over 3 years of follow-up.

### PBMC sample collection, RNA isolation, microarray hybridization, and data processing

See details in Additional file 1*.* Microarray experiments were compliant with MIAME (Minimum Information About a Microarray Experiment) guidelines. The complete data sets are available in the Gene Expression Omnibus database (http://www.ncbi.nlm.nih.gov/geo/) under accession number GSE28221.

### Identification of gene co-expression modules correlated with clinical traits in training cohort

Normalized microarray data were filtered to remove redundant genes and genes with minimum variation (i.e. coefficient of variation <0.3 across all samples). Genes that passed filtering criteria were clustered into gene modules, based on their co-expression pattern, using an unsupervised “Weighted gene co-expression network analysis (WGCNA)” package in R 2.13 [23]. Principal Component Analysis (PCA) was used to calculate an eigengene for each gene module. Pearson’s correlation was used to determine the significance of correlation (*p* < 0.05) between the eigengenes of individual gene modules with each clinical parameter including race, sex, age, FVC % predicted, D_L_CO % predicted, and CPI.

### Identification of differentially expressed genes in the training cohort

Significant Analysis of Microarray (SAM) software [24] was used to identify differentially expressed genes between observed good vs. poor IPF prognosis using criteria of fold change (FC) >1.5 and false discovery rate (FDR) < 2 %.

### Survival analysis

Survival analysis was performed using unadjusted log rank testing along with univariate and/or multivariate Cox regression analysis. After checking to ensure that the proportional hazard assumption was met with each Cox model, subdistributional mortality hazards were determined for covariates by treating lung transplantation as a competing event, as previously described by Fine and Gray [25]. Survival time was defined as time from blood draw to death, transplant, loss-to-follow-up or study conclusion. Patients who were lost to follow-up were censored at that time in survival modeling. Survival between groups was plotted using the Kaplan-Meier estimator.

### Compilation of the IPF prognostic predictor gene set from the training cohort for a genomic model construction

To construct a functional genomic model predictive of IPF prognosis, genomic, clinical, and outcome data from the training cohort were analyzed to identify a set of genes with individual prognostic significance. Genes were selected for the “IPF prognostic predictor gene set” if they met all of the following criteria: 1) genes in specific gene co-expression modules that correlated with pulmonary function (*p* < 0.05) in WGCNA, 2) genes differentially expressed (FC > 1.5 and FDR < 2 %) between observed good vs. poor prognosis by SAM, and 3) genes predictive of mortality (*p* < 0.05) in univariate Cox regression analysis.

### Development and validation of the functional genomic model to predict prognosis

The set of IPF prognostic predictor genes identified was used to construct a genomic model using “Survival risk group prediction” implemented in BRB-ArrayTools 4.2 [26] to predict prognosis in IPF patients. The output of the genomic model is a patient-specific “prognostic index (PI)” score. PI of each patient in training cohort was derived from formula, ∑W_*i*_ *  X_*i*_ + 13.5, where *W*_*i*_ and *X*_*i*_ represent the weight (computed by supervised PCA) and log-intensity of the *i-*th gene in the gene set. To assign a patient to either a high- or low-risk group, each patient’s PI was compared to a predetermined classification threshold. For this study, the threshold was set at the upper tertile in the training cohort according to clinical observation [3]. A “10-fold Cross-Validation (CV)” algorithm was used to assess the classification specificity. Briefly, 10 % of patients were randomly omitted leaving the remaining 90 % of patients to construct the genomic model and derive a PI for each of the omitted samples. The PI of omitted individuals was then ranked relative to the PI of patients included in the CV model. Finally, we determined the predicted risk category based on the percentile ranking, the number of risk groups specified (i.e. *n* = 2 in current study), and the empirical risk percentile setting (i.e. low/high risk = 66.7/33.3). Misclassification rate was determined by the discrepancy between the predicted low or high-risk category with the observed good or poor prognosis according to follow-up. Receiver-Operating-Characteristic (ROC) analysis with area under curve (AUC) calculation was performed to assess how well the PI distinguished IPF patients with low vs. high-risk prognosis. To perform an independent validation of the predictor, we applied the PI weights computed from the training set of 45 IPF samples to the calculation of the PI on the UCV and UPV cohorts. Details can be found in Additional file 1.

### Functional pathways enrichment analysis

Significant biological processes in Gene Ontology associated with the set of IPF prognostic predictor genes were identified using R package “GOSim” [27] with the criterion of *q*-value (Benjamini-Yekutieli adjusted *p*-value) <0.01. Significant canonical pathways or gene interaction networks were analyzed using Ingenuity Pathway Analysis (IPA) software (Ingenuity Systems, Redwood City, CA) with the criterion of the right-tailed (referring to the overrepresented pathway) Fisher’s exact test *q*-value (Benjamini-Hochberg adjusted *p*-value) <0.05.

### IPF diagnosis prediction using prognosis index derived from the functional genomic model

Using the generated PI, ROC analysis with AUC calculation was performed in UCV cohort to assess how well the PI distinguishes IPF patients from healthy controls. The true positive rate (sensitivity) is plotted in function of the false positive rate (1-specificity) for different cut-off points. Each point on the ROC curve represents a sensitivity/false alarm pair corresponding to a particular decision threshold.

### Statistical analysis

Continuous variables are reported as a mean (± standard deviation) and compared using a one-way analysis of variance. Categorical variables are reported as counts and percentages and compared using a chi-square or Fischer’s exact test, as appropriate. Pearson’s correlation was used to evaluate the correlation of prognostic index (PI) derived from genomic model with clinical parameters. ROC analysis with AUC calculation was performed using R package “caTools”. Other than when indicated above, statistical analysis was conducted using STATA 12 (StataCorp. 2011. College Station, TX).

## Results

### Demographic and clinical characteristics of patients with IPF

Demographic and clinical characteristics for each IPF cohort are shown in Table 1. Significant differences between the training, UCV and UPV cohorts were observed with respect to male gender (90 % vs. 71.4 % vs. 69.3 %, respectively; *p* = 0.05), white race (82.2 % vs. 81.8 % vs. 97.3 %, respectively; *p* = 0.004), follow-up months (18.8 vs. 43.8 vs. 23.5 months, respectively; *p* < 0.001), months to death (12.7 vs. 26.8 vs. 14.2, respectively; *p* = 0.02) and lung transplantation (2.2 % vs. 9.5 % vs. 20 %, respectively; *p* = 0.009). No differences between cohorts were observed with respect to age, FVC % predicted, DLCO % predicted or CPI.

**Table 1:** Demographic and Clinical Characterizations among Study Cohorts

Characteristic	Training cohort (*n* = 45)	UCV cohort (*n* = 21)	UPV cohort (*n* = 75)	*p*-value
Age, mean (±SD)	67.1 (8.2)	68.9 (8.2)	68.5 (7.8)	0.48
Male gender, *n* (%)	40 (90)	15 (71.4)	52 (69.3)	0.05
White race, *n* (%)	37 (82.2)	18 (81.8)	73 (97.3)	0.004
Follow-up months, mean (±SD)	18.8 (11.9)	43.8 (29.4)	23.5 (12.7)	<0.001
Months to death, mean (±SD)	12.7 (10.9)	26.8 (20.1)	14.2 (10.6)	0.02
FVC % predicted, mean (±SD)	60.6 (14.3)	64.7 (12.7)	65.4 (16.7)	0.25
DLCO % predicted, mean (±SD)	43.4 (17.7)	43.2 (15.6)	48.9 (18.6)	0.19
CPI, mean (±SD)	55.6 (13)	54.7 (10.7)	50.7 (13.7)	0.11
Lung transplantation, *n* (%)	1 (2.2)	2 (9.5)	15 (20)	0.009

### Identification of gene co-expression modules correlated with clinical traits in training cohort

**Fig. 1:**
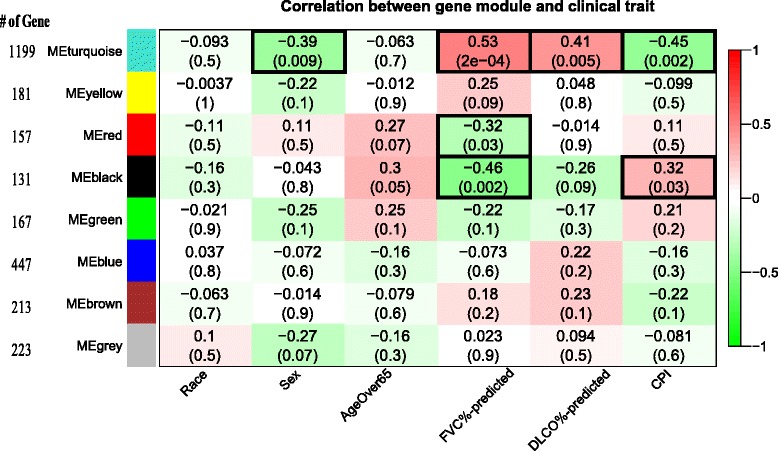
Correlation of gene co-expression modules with clinical traits in training cohort (*n* = 45). Gene co-expression modules were constructed using R package WGCNA (see Methods *and* Additional file 1 for detail), and denoted by different colors. The parameters for topological overlap matrix generation and unsupervised gene clustering are displayed in Additional file 3: Figure S2A & S2B. The number of genes in each gene module is labeled on left. The module eigengene is the principal component of each gene module computed across all samples. Correlation of module eigengene with each clinical trait was determined by Pearson’s correlation algorithm and displayed in the corresponding box (coefficient on top and *p*-value in parenthesis on bottom). The color of each box represents the direction of correlation (red) or anti-correlation (green) and the degree of correlations are scaled by the bar on the right. Traits significantly associated with specific modules are highlighted with a purple frame. FVC % predicted = forced vital capacity percent predicted; D_L_CO % predicted = diffusion capacity of carbon monoxide percent predicted; CPI = composite physiologic index

“WGCNA” package in R was utilized to cluster 2718 genes that passed the filtering criteria into eight gene co-expression modules denoted by different colors (Fig. 1). Optimization of the power for adjacency transition and the parameters for gene clustering dendrogram are depicted in Additional file 2: Figure S1A and S1B, respectively. The eigengene values of individual modules were then computed by PCA and correlated with clinical traits to envision the association between co-expressed gene pattern features with clinical features [23]. The significance of correlation with clinical traits was determined by Pearson’s correlation assay with *p* <0.05. As shown in Fig. 1, three gene modules demonstrated significant correlation (red box) or anti-correlation (green box) with clinical traits: turquoise module with male gender (*p* = 0.009), FVC % predicted (*p* = 0.0002), D_L_CO % predicted (*p* = 0.005), and CPI (*p* = 0.002); red module with FVC % predicted (*p* = 0.03); black module with FVC % predicted (*p* = 0.002) and CPI (*p* = 0.03). Of note that 1199, 157, and 131 genes consist of 55 % (1487/2718) of total genes were clustered into turquoise, read, and black modules, respectively (Fig. 1).

### Compilation of the set of IPF prognostic predictor genes

**Fig. 2:**
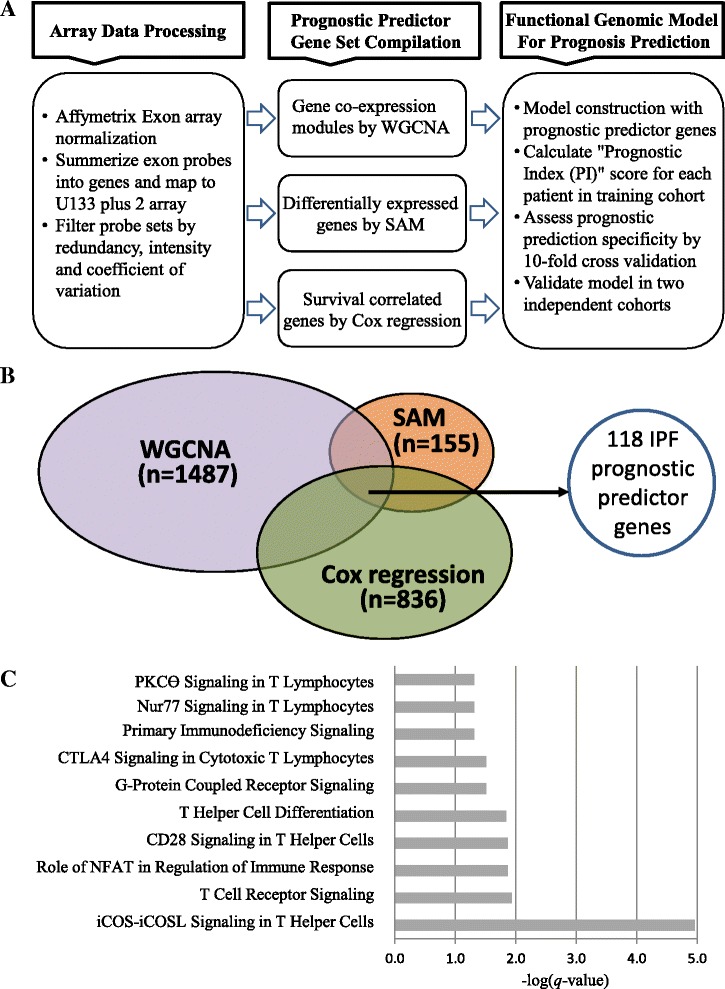
Compilation and functional characterization of IPF prognostic predictor gene set. **a** A flowchart illustrates the procedures and approaches used for IPF prognostic predictor gene set compilation. *Left panel: Arrary data processing.* Affymetrix Exon 1.0 ST Array data was normalized, probe sets mapped to U133 plus 2 Array, and filtered based on redundancy, intensity, and coefficient of variation across all samples. *Middle panel: IPF prognostic predictor gene set compilation.* Three approaches used to compile IPF prognostic predictor gene set: Co-expressed gene modules correlated with pulmonary function identified by WGCNA; Differentially expressed genes between “good” and “poor” prognosis patients identified by SAM (fold change > 1.5 & FDR < 2.5 %); Survival-correlated genes identified by Cox regression (*p* < 0.005). *Right panel*: *Genomic model IPF prognosis prediction*. IPF prognostic predictor gene set was used to construct a genomic model; Prognostic Index (PI) score was calculated from each patient in training cohort; Prediction specificity was assessed by 10-fold cross validation; Genomic model was validated in two independent cohorts using weights of PI calculated from training cohort. **b** Venn diagram illustrates the selection criteria for IPF prognostic predictor genes. A total of 118 genes were compiled for downstream data analyses. **c** Canonical pathways enriched from IPF prognostic predictor genes by Ingenuity Pathway Analysis software. Significant pathways were set with criterion of *q*-value < 0.05 (i.e. -log (*q*-value) > 1.3) using one-tailed Fisher’s exact test. X-axis represents -log (*q*-value)

In an effort to reduce the number of genes in turquoise, red, and black modules associated with pulmonary function to a clinically applicable number and to incorporate predictive prognostic feature to co-expressed gene pattern feature, we applied two additional distinct approaches to the analytic pipeline (Fig. 2a). Using SAM, we identified 155 that were differentially expressed (DE) genes between IPF patients with good and poor prognosis. Using univariate Cox regression analysis, we identified 836 genes which were significantly correlated with survival (*p* < 0.05) (Fig. 2b). Notably, 147 of the 155 DE genes were overlapped with the 1487 genes combined from the turquoise, black, and red gene modules. This integrative functional genomic approach yielded a set of 118 prognostic predictor genes (Fig. 2b). A list of 118 prognostic predictor genes attributed to turquoise (*n* = 110), red (*n* = 5), and black (*n* = 3) modules was shown in Table 2.

**Table 2:** List of 118 IPF prognostic predictor genes within the red, black and turquoise gene modules

Gene	FC	Gene	FC	Gene	FC	Gene	FC
IL1R2^¥^	2.0	PPWD1	−1.7	ASF1A	−1.6	ABCD2	−1.5
ERAF^§^	2.0	CETN3	−1.6	LMO7	−1.6	GZMK	−1.5
CEACAM8^¥^	1.8	SH2D1A	−1.6	GCET2	−1.6	TRIM52	−1.5
ARG1^¥^	1.6	SLC39A10	−1.6	PAQR8	−1.6	C8orf15	−1.5
FOXO3^§^	1.5	SHPRH	−1.6	BIRC3	−1.6	ITK	−1.5
TNS1^§^	1.5	WDR75	−1.6	CAMK4	−1.6	ICOS	−1.5
CYP4F2^¥^	1.5	C14orf64	−1.6	ZC3H6	−1.6	FHIT	−1.5
CYP4F3^¥^	1.5	KPNA5	−1.6	CD28	−1.6	TSEPA	−1.5
ARHGAP5	−1.8	NOP58	−1.6	GTPBp0	−1.6	NPCDR1	−1.5
ORC3L	−1.8	PARp5	−1.6	C5orf51	−1.6	OXNAD1	−1.5
ZNF100	−1.8	PRO0471	−1.6	TRBC1	−1.6	IL7R	−1.5
UTp5	−1.8	RCAN3	−1.6	CAMK2D	−1.5	HLA-DQA1	−1.5
ANKRD36B	−1.8	C7orf64	−1.6	PPM1K	−1.5	TMEM156	−1.5
LOC399753	−1.8	ANKRD36	−1.6	CCDC76	−1.5	HLA-DQA1	−1.5
KCNA3	−1.8	GPR174	−1.6	CASD1	−1.5	LOC401397	−1.5
RHOH	−1.8	NDUFAF4	−1.6	pRY10	−1.5	CDK6	−1.5
LCK	−1.8	CCDC141	−1.6	DPP4	−1.5	GCNT4	−1.5
C16orf52	−1.7	GPR18	−1.6	S1PR1	−1.5	NELL2	−1.5
TC2N	−1.7	DDX60	−1.6	ITGA6	−1.5	FLJ33630	−1.5
HIVEp	−1.7	TMEM209	−1.6	GBP4	−1.5	TRAT1	−1.5
KIF3A	−1.7	GVIN1	−1.6	ABCE1	−1.5	LEF1	−1.5
IFT80	−1.7	TMEM161B	−1.6	TXK	−1.5	FCRL3	−1.5
TIA1	−1.7	USP53	−1.6	TRAF5	−1.5	GUSBL2	−1.5
ZNF83	−1.7	TRAJ17	−1.6	SLAMF6	−1.5	SEPSECS	−1.5
SETDB2	−1.7	MRPL1	−1.6	CD96	−1.5	BTLA	−1.5
WDR36	−1.7	SNORD116	−1.6	PRKACB	−1.5		
ZNF141	−1.7	GPR171	−1.6	ALG10B	−1.5		
TRBC1	−1.7	MGC40069	−1.6	NBPF10	−1.5		
FAM69A	−1.7	LOC439949	−1.6	MGAT4A	−1.5		
C1GALT1	−1.7	CCR7	−1.6	INPP4B	−1.5		
GIMAP5	−1.7	NUP43	−1.6	STAT4	−1.5		

FC = Fold change; ^¥^denotes genes in red module; ^§^denotes genes in black module; the rest of genes are in turquoise module

### Pathway and network characterization of the IPF prognostic predictor genes

To assess the pathways and networks of the 118 IPF prognostic predictor genes involved, we carried out a functional enrichment analysis using Ingenuity pathway analysis (IPA) and gene network analysis software. Surprisingly, all significant canonical pathways with -log(*q*-value) > 1.3 were involved in T-cell biology (Fig. 2c). Several genes were involved in multiple T-cell signaling pathways, including CD28 receptor (*CD28),* inducible T-cell co-stimulator *(ICOS),* lymphocyte-specific protein tyrosine kinase (*LCK),* interleukin 7 receptor *(IL7R),* and major histocompatibility complex, class II, DQ alpha 1 (*HLA-DQA1)* (Additional file 1: Table S1). Of note that these pathways comprised genes mostly if not all in the turquoise module which represent >93 % of genes in the IPF prognostic predictor gene set.

Ingenuity network modeling based on “Ingenuity Knowledge” database prioritized five significant gene networks with score ≥ 32. Seventy-eight of the 118 IPF prognostic predictor genes (66 %) was functionally connected in these five gene networks, supporting a concordance between expression correlations and functional connections of individual genes. The first of the five gene interaction networks is displayed in Additional file 3: Figure S2 showing that five hub genes have significantly higher degree of linkage to other nodes in the network including ras homolog gene family member H (*RHOH)*, G protein-coupled receptor 18 (*GPR18)*, G protein-coupled receptor 171 (*GPR171)*, and G protein-coupled receptor 174 (*GPR174)*, and lymphocyte-specific protein tyrosine kinase (*LCK*).

### Construction and cross-validation of a functional genomic model for prognosis prediction

**Fig. 3:**
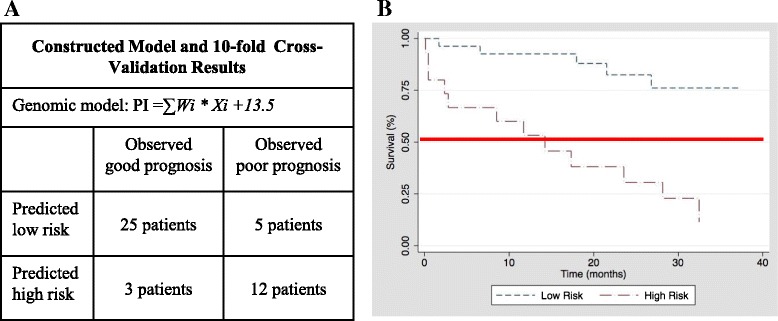
Genomic model and 10-fold cross validation results. **a** A genomic model was constructed from the 118 IPF prognostic predictor genes using “Survival risk group prediction” algorithm implemented in BRB-ArrayTools (see Additional file 1) followed by 10-fold cross validation (CV) algorithm to calculate the misclassification rate. Formula of genomic model: Prognostic index (PI) = ∑W_*i*_ * X_*i*_ +13.5, where *W*_*i*_ and *X*_*i*_ represent the weight and log-intensity of the *i-*th gene in IPF prognostic predictor gene set compiled from training cohort, respectively. Misclassification rate (20 %) was determined by 10-fold CV and computed as (k + n)/total cases, where k represents the predicted high risk that are observed as good prognosis, and n represents the predicted low risk that are observed as poor prognosis. **b** IPF patients with predicted low (dotted line) and high risk (dashed line) stratified by prognostic index (PI) derived from each patient in training cohort based on the genomic model. The red line denotes 50 % probability of survival. PI independently predicted survival in univariate competing-risk Cox regression (Sub-hazard ratio (SHR) 2.7; 95 % CI 1.9-3.9; *p* < 0.001) and in multivariate competing-risk Cox regression after adjustment for baseline CPI (SHR 2.3; 95 % CI 1.5-3.4; *p* < 0.001)

To determine prognosis prediction power of the gene set, we constructed a novel functional genomic model using “Survival risk group prediction” implemented in BRB-ArrayTools. The output of the genomic model is a patient-specific prognostic index (PI). The genomic model was displayed in Fig. 3a, where *W*_*i*_ and *X*_*i*_ represented the weight and log-intensity of the *i-*th gene in the gene set identified from training cohort. Based on previous clinical observation, we empirically set the percentile population of low vs. high risk to the lower tertiles vs. upper tertile, respectively. Patients in the training cohort were categorized as low or high risk based on whether the patient-specific PI fell in the lower tertiles vs. upper tertile, respectively. We obtained PI values ranging from −2.14 to 3.45, which were continuously associated with risk of death.

Of the 45 individuals in the training cohort, 30 (66.7 %) and 15 (33.3 %) were assigned to the low risk or high risk groups, respectively based on PI. Misclassification rate was determined by comparison of the predicted class to clinically observed outcomes in the training cohort, in which 28 patients were with “good” and 17 with “poor” prognosis (Fig. 3a). Ten-fold cross-validation (CV) demonstrated a low misclassification rate of 20 %. In order to estimate the variance, we repeated the 10 fold CV 10 times with random partitions of the training cohort. The range of misclassification was 17 % ~ 23 %. Survival was significantly better among those classified as low risk (*p* < 0.001 compared to high risk) based on PI score (Fig. 3b). PI independently predicted survival in univariate competing-risk Cox regression (Sub-hazard ratio (SHR) 2.7; 95 % CI 1.9-3.9; *p* < 0.001) and in multivariate competing-risk Cox regression after adjustment for baseline CPI (SHR 2.3; 95 % CI 1.5-3.4; *p* < 0.001).

### IPF genomic model predicts prognosis in two independent validation cohorts

**Fig. 4:**
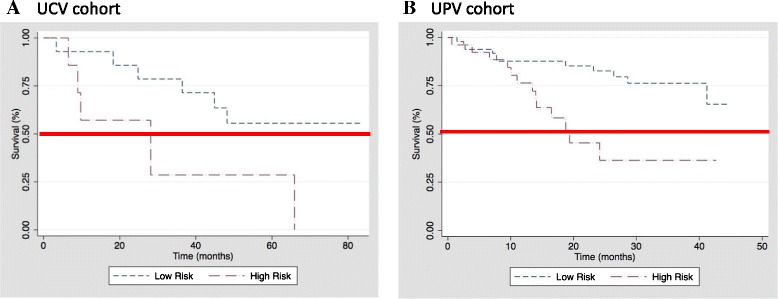
IPF genomic model predicts prognosis in two independent validation cohorts. The prognosis prediction specificity was assessed in University of Chicago validation cohort (UCV, panel **a**) and University of Pittsburgh validation cohort (UPV, panel **b**). IPF patients with predicted low (dotted line) and high risk (dashed line) stratified by prognostic index (PI) derived from each patient in UCV and UPV cohorts based on the genomic model. The red line denotes 50 % probability of survival. PI significantly predicted survival in univariate competing-event Cox regression in the UCV (SHR 2.0; 95 % CI 1.2-3.4; *p* = 0.005) and UPV (SHR 1.8; 95 % CI 1.1-2.7; *p* = 0.01) cohorts. This association remained in the UCV (SHR 1.7; 95 % CI 1.04-2.93; *p* = 0.035) and UPV (SHR 1.9; 95 % CI 1.2-3.0; *p* = 0.005) cohorts after adjusting for baseline CPI in multivariate Cox regression

The IPF genomic model shown in Fig. 3a was applied to two independent validation cohorts, UCV and UPV, where two microarray platforms (Affymetrix and Agilent, respectively) were used. The weight of each gene and the constant (13.5) derived from training cohort were carried over for independent validation of the genomic model. After annotating the gene expression data from each cohort with UniGene annotations, 10 of the IPF prognostic predictor genes did not map to Agilent Human 4x44k Whole Genome Expression array in UPV. Therefore, we computed the PI using 108 classifiers for UPV and 118 classifiers for UCV, respectively. Individuals in the UCV and UPV cohorts were classified as low vs. high risk based on whether the patient-specific PI fell in the lower tertiles vs. upper tertile, respectively, as was done for the training cohort. In both validation cohorts, patients classified as low risk demonstrated significantly improved survival over those classified as high risk (*p* < 0.001 for UCV and *p* = 0.002 for UPV) (Fig. 4a&b). PI remained a significant predictor of survival in univariate competing-event Cox regression in the UCV (SHR 2.0; 95 % CI 1.2-3.4; *p* = 0.005) and UPV (SHR 1.8; 95 % CI 1.1-2.7; *p* = 0.01) cohorts. This association remained in the UCV (SHR 1.7; 95 % CI 1.04-2.93; *p* = 0.035) and UPV (SHR 1.9; 95 % CI 1.2-3.0; *p* = 0.005) cohorts after adjusting for baseline CPI in multivariate Cox regression.

To evaluate the biological reproducibility of IPF prognostic predictor genes within the set, fold change of each gene was calculated between predicted low-risk and high-risk prognosis patients in each cohort, and then regressed between training versus each validation cohort. The regression plot revealed a strong concordance of fold changes between training and each validation cohort (Additional file 4: Figure S3). Pearson’s correlation analysis showed significant correlation of the classifier fold changes between training and UCV (*p* = 2.2 × 10^−16^) as well as between training and UPV cohort (*p* = 1.4 × 10^−8^).

### Multivariate correlation of Prognostic Index with clinical parameters

We investigated the impact of clinical parameters on the genomic model (Additional file 1: Table S2). Pearson’s correlation analysis showed that PI was significantly correlated with CPI in the training (coefficient = 0.36, *p* = 0.016) and UCV (coefficient = 0.53, *p* = 0.01) cohorts, but not in UPV cohort (coefficient = −0.05, *p* = 0.69). While the PI of an individual sample was not associated with age, it was significantly higher in males compared to females in the training and UPV cohorts (*p* = 0.02 and 0.04, respectively). The impact of ethnic diversity on PI was evaluated and no difference was found between Caucasian and non-Caucasian in the training (*p* = 0.92) and UCV cohort (*p* = 0.79) despite each cohort containing 18 % non-Caucasians (Additional file 1: Table S2). This result was consistent with the data obtained from WGCNA analysis showing the turquoise gene module was significantly correlated with female gender (Fig. 1, coefficient = −0.39, *p* = 0.009).

### Application of genomic model in IPF diagnosis

To evaluate whether our genomic model is able to discriminate IPF patients from healthy individuals, we conducted the ROC analysis of the PI by plotting the true positive (sensitivity) versus false alarm (1-specificity). PI accurately distinguished IPF subjects from healthy individuals in the UCV cohort with an AUC 0.96. The sensitivity of IPF diagnosis by PI at 10 % false alarm was 80 % (red line in Additional file 5: Figure S4).

## Discussions

In this study, we constructed a functional genomic model that predicted survival in three independent cohorts of IPF patients. In the training cohort, we analyzed genomic data using both unsupervised WGCNA and supervised SAM approaches. By applying WGCNA algorithm, we first associated the pathophysiological alterations in the transcriptome level to the clinic traits of IPF and found 55 % of the genes clustered into the turquoise, black and red modules which were significantly correlated with pulmonary function. In a parallel analysis, 95 % of the differentially expressed genes between IPF patients with good and poor prognosis identified by SAM were attributed to these three pulmonary function associated gene modules. This analytical pipeline highlights the potential applicability of an unsupervised correlation network approach, whereby functional characterization of correlated gene modules provides insight into the molecular mechanisms underlying a clinical trait of a complex pulmonary disease. Lastly, we correlated gene expression levels with survival, which contributed another important feature of IPF. We defined genes met all three selection criteria as “IPF prognostic predictor genes”.

Pathway analysis of the IPF prognostic predictor genes revealed several canonical pathways including T-cell receptor signaling pathway in turquoise module (*q* = 0.0087); hemoglobin metabolic process and oxygen transport in black module (*q* = 0.0031 and 0.0032, respectively); and defense response to bacterium and neutrophil degranulation in red module (*q* = 0.000 and 0.0041, respectively) (Additional file 1: Table S3). The enriched T-cell biology, including iCOS signaling in T-helper cells, CD28 signaling, and T-helper cell differentiation is supported by our prior work, with similar analyses of a smaller gene set demonstrating that decreased expression of CD28, ICOS, LCK, and ITK predicted mortality in patients with IPF [16]. Impaired regulatory T-cells from bronchoalveolar lavage fluid have been strongly correlated with pulmonary dysfunction of IPF patients [28]. Down-regulation of CD28 on circulating CD4 T-cells has been associated with poor outcomes in IPF patients [29]. IL-17A, a cytokine produced by CD4^+^ and gamma-delta^+^ T cells, has been shown to play a critical role in inducing fibrosis in a mouse model [30]. Although the role of the immune system in IPF remains unclear, a large multicenter study has shown that IPF patients treated with prednisone and azathioprine had an increased risk of death and hospitalization compared to those receiving placebo [31]. It remains unknown whether a down-regulated immune system is causally involved in IPF pathogenesis, or is the result of primary lung injury. In addition, a down-regulated immune system could result in a reduced T-cell population [32, 33]. Nevertheless, the down-regulated T-cell pathways or reduced T-cell population can both lead to impaired immune function. These studies are congruent with the functional profile of our IPF genomic model suggesting that suppression of the immune system with medications such as prednisone and azathioprine may worsen the clinical course for IPF patients whose immune systems are already down-regulated.

By evaluating the performance of the genomic model in two independent validation cohorts with different microarray platforms performed at different medical centers, we demonstrate the potential applicability of our findings for real-world use. Notably, the prognostic index (PI) derived from the genomic model showed consistent prognostic prediction specificity in each validation cohort and produced similar mortality hazard estimation across all three cohorts. Genomic model constructed using IPF prognostic predictor genes also displayed concordant fold changes between patients with predicted low- and high-risk prognosis in training and validation cohorts. Furthermore, the PI was able to discriminate between IPF and healthy controls with great accuracy, suggesting a future potential screening tool. However, it is unclear whether the PI can distinguish IPF patients from patients with other pulmonary fibrotic diseases such as nonspecific interstitial pneumonia (NSIP), hypersensitivity pneumonitis (HP), and respiratory bronchiolitis-associated interstitial lung disease (RB-ILD) etc. This question can be addressed in future studies.

While the genomic model developed in this study has been successfully validated in two independent (UCV and UPV) cohorts, certain technical issues and potential clinical confounders require further study. First, there were demographic differences in gender and race between the training and UPV cohorts. The training population was strongly biased towards male patients, while the UPV population was more balanced with respect to gender. There was a greater prevalence of Caucasians in the UPV cohort. Interestingly, the PI scores were higher and indicative of poorer outcome in women overall, while being primarily derived from a male cohort. While IPF is more common in men than women [31], women appear to have improved survival [34, 35]. Although the reasons for this clinical observation are unclear, our results indicate that there may be differential gene expressions between male and female patients with IPF that underlie this observation.

In addition, the different microarray platforms used in different cohort studies might affect the prediction specificity of the model. Notably, the correlation of the PI with pulmonary function in UPV cohort is less strong compared to that in training and in UCV cohorts. We speculated that this observation may be partially attributed to the loss of the 10 classifiers when mapped from Affymetrix to Agilent microarray platform. Another potential confounding factor is the higher rate of lung transplant in the UPV cohort (20 %) compared to the UCV cohorts (7 %). We attempted to adjust for this in our survival analysis by treating transplant as a competing event. Validation with a larger prospective cohort would be beneficial. Finally, the PI cannot be standardized across different microarray platforms, because the gene expression levels in microarray assay were measured by arbitrary fluorescent intensities rather than transcript copy numbers. Therefore, an absolute cut-off or carry-over of PI across individual studies is not feasible, and clinical elaboration of the hazard ratio of PI is impractical at this stage. Future approaches with direct assessment of these IPF prognostic predictor genes could overcome this issue.

## Conclusions

We identified an IPF genomic model with both diagnostic and prognostic prediction ability. The unsupervised WGCNA analysis appears to be a promising approach to elucidate the molecular mechanism underlying IPF progression as an extension of its previous use in oncologic studies [36–38]. The genomic model constructed from the IPF prognostic predictor genes demonstrated robust clinical applications. Functional analysis of the IPF prognostic predictor genes strongly supported the involvement of T-cell immune response in IPF progression [29, 39]. These data continue to support and highlight the use of genomic profiles from the peripheral blood for pulmonary disease.

## Additional files

Additional file 1:
**Additional Methods and Tables.** (DOCX 103 kb) Additional file 2: Figure S1. Detection of gene co-expression modules in training cohort. Gene expression intensities obtained from Exon 1.0 ST Array were normalized. Probe sets were mapped to U133 plus 2.0 Array and filtered as described in Additional file 1. A total of 2,718 unique genes were retained and subjected to R package “Weighted Gene Co-expression Network Analysis (WGCNA)” to identify co-expressed gene modules. A). Optimization and selection of power for adjacency transition of gene-gene correlation matrix (power =7). B). Cluster dendrogram of the gene co-expression modules represented by different colors. Seven gene co-expression modules were detected by hierarchical clustering using dynamic tree cut algorithm integrated in WGCNA with the following parameters: power=7, minModuleSize=120, mergeCutHeight= 0.3. Unclustered genes (genes not correlated with other genes) were collected in Grey module. (PPTX 187 kb) Additional file 3: Figure S2. Gene interaction network of IPF prognostic predictor genes. Significant gene interaction networks were determined using Ingenuity Pathway Analysis (IPA) software. Node shapes denoting different functions were depicted in right panel box. Green and red denote down and up-regulated genes, respectively. (PPTX 544 kb) Additional file 4: Figure S3. Concordance of IPF prognostic predictor genes between training and each validation cohort. The fold change of each gene between predicted low-risk and high-risk prognosis patients was plotted between training (X-axis) and validation cohort (Y-axis). (PPTX 51 kb) Additional file 5: Figure S4. Receiver-Operating-Characteristic (ROC) analysis of genomic model for diagnosis prediction. ROC curves of UCV cohort consisting of IPF patients and healthy individuals were plotted based on the Prognostic Index (PI) derived from IPF genomic model. AUC (Area-Under-Curve) is displayed in the graph. The red line denotes 10 % false alarm (1-Specificity). (PPTX 79 kb)

## Abbreviations

IPF: idiopathic pulmonary fibrosis
PBMC: peripheral blood mononuclear cells
WGCNA: weighted gene co-expression network analysis
SAM: significance analysis of microarray
SHR: sub-hazard ratio
ROC: Receiver-Operating-Characteristic analysis
AUC: Area-Under-Curve
PI: prognostic index
PCA: principal component analysis
UCMC: University of Chicago Medical Center
UPMC: University of Pittsburg Medical Center
UCV: University of Chicago validation cohort
UPV: University of Pittsburgh validation cohort
FVC % predicted: forced vital capacity percent predicted
D_L_CO % predicted: diffusion capacity of carbon monoxide percent predicted
CPI: composite physiologic index
FC: fold change
FDR: false discovery rate
CV: cross validation
IPA: ingenuity pathway analysis
COMET: correlating outcomes with biochemical markers to estimate time-progression in IPF
NSIP: conspecific interstitial pneumonia
HP: hypersensitivity pneumonitis
RB-ILD: respiratory bronchiolitis-associated interstitial lung disease
