# Supplementary material for: A functional genomic model for predicting prognosis in idiopathic pulmonary fibrosis
Source: BMC Pulm Med. 2015 Nov 21;15:147. doi: 10.1186/s12890-015-0142-8 (PMC4654815; doi:10.1186/s12890-015-0142-8)
Supplement: Additional file 1: — Additional Methods and Tables. (DOCX 103 kb) [file 12890_2015_142_MOESM1_ESM.docx]

**Additional file 1**

**A functional genomic model for predicting prognosis in idiopathic pulmonary fibrosis**

Yong Huang^1^*, Shwu-Fan Ma^1^*, Rekha Vij^1^*, Justin M Oldham^1^, Jose Herazo-Maya^2^, Steven M Broderick^1^, Mary E Strek^1^, Steven R White^1^, D Kyle Hogarth^1^, Nathan K Sandbo^1^, Yves A Lussier^3,4^, Kevin F Gibson^5^, Naftali Kaminski^2^, Joe GN Garcia^6^, Imre Noth^1#^

*These authors contributed equally

^1^Section of Pulmonary & Critical Care Medicine, University of Chicago, Chicago IL; ^2^Pulmonary, Critical Care and Sleep Medicine, Yale University, New Haven, CT;^3^Institute for Genomics and Systems Biology, University of Chicago, Chicago IL; ^4^Department of Medicine, Bio5 Institute, UA Cancer Center, University of Arizona, Tucson, AZ; ^5^Division of Pulmonary, Allergy and Critical Care Medicine, University of Pittsburgh, Pittsburgh, PA; ^6^Arizona Respiratory Center and Department of Medicine, The University of Arizona, Tucson, AZ

**Additional methods**

**UCMC sample collection, RNA isolation, microarray hybridization and data processing.** Total RNA was re-precipitated by sodium acetate/ethanol, further processed for microarray assay using Affymetrix Human Exon 1.0 ST GeneChip (Santa Clara, CA).

RNA quality and integrity were confirmed by Nanodrop (A260/A280 ratios between 1.7 and 2.2) and Bio-Analyzer mini-gel assay. In each PBMC sample, 150ng RNA was adopted for reverse transcription to single stranded DNA, and amplification of cRNA using Affymetrix GeneChip WT cDNA Synthesis Kit according to GeneChip Expression analysis technical manual (S1). Amplification yields of cRNA for the first and second rounds (exceeding 25 μg/ml and 1000 μg/ml, respectively), and quality of the second round amplified cRNAs were all satisfactory. Hybridization and scanning were performed as described in the GeneChip Expression Manual (S1).

The microarray raw data (.cel files) were processed using dChip software with the following parameters: Model method="average", Normalization="quantile", Smoothing method="running median". The probe grouping file "HuEx-1_0-st-v2.r2.pgf" and probe set mapping file "U133_to_exon_mapping.csv" were downloaded from Affymetrix website. The intensities of all exons in a gene were averaged based on probe grouping file to represent the corresponding gene expression value, and further mapped to Affymetrix U133 probe sets based on consensus sequences specified in "U133_to_exon_mapping.csv" file. The filtering steps applied to U133 probe sets included: 1) removing probe sets without functional annotations; 2) for redundant probe sets targeting on the same gene, only keeping the one with the highest mean values across all samples; and 3) removing probe sets with only low expression intensities or minimal variation across samples (intensity < 100 in more than 70% samples or coefficient of variation <0.3 across all samples). A total of 2,718 unique genes passed these criteria in training cohort and were adopted for downstream data analysis.

**Construction and cross-validation of genomic model for IPF prognostication**. A genomic prognostication model with IPF prognostic predictor genes prioritized from training cohort was constructed using BRB-ArrayTools [S2]. The number of risk groups and the risk percentiles were specified by user. The "Survival risk group prediction" algorithm in BRB-ArrayTools uses a Cox-PH model to relate survival time to k “super-gene” expression levels (we set k =2 in current study). The “supergene” expression levels are the k Principal components, i.e. linear combinations of expression levels of the preselected specified classifiers [S3]. The k-variable Cox-PH regression analysis was performed. This provides a regression coefficient (weight) for each principal component. The average of the coefficients of the k components was defined as the weight of the corresponding classifier. To compute a prognostic index (PI) for a patient, the software provided a prognostic index (PI) with a log expression intensity given by a vector x. A high value of the PI corresponds to a high value of hazard or risk, and consequently a relatively poor predicted prognosis.

Ten-fold cross-validation (CV) was used to evaluate the misclassification rate of the genomic model in training cohort. Briefly, PI of the 10% of the randomly omitted patients was ranked relative to the PI of the rest of the 90% of the patients included in the model. The omitted patient was placed into a risk group based on his/her percentile ranking, the number of risk groups specified, and the cut-off percentiles specified for defining the risk groups. This analysis was repeated from scratch n times (n=10), leaving out a different 10% of patients each time.

**Additional References:**

S1. <http://media.affymetrix.com/support/downloads/manuals/expression_analysis_>

technical_manual.pdf

S2. Simon R, Lam A, Li MC, Ngan M, Menenzes S, et al. (2007) **Analysis of gene expression data using BRB-ArrayTools.** *Cancer Inform* **3:** 11-17.

S3. Bair E, Tibshirani R. (2004) **Semi-supervised methods to predict patient survival from gene expression data.** *PLoS Biol* **2:** E108.

**Additional figure legends**

**Additional Figure S1. Detection of gene co-expression modules in training cohort.** Gene expression intensities obtained from Exon 1.0 ST Array were normalized. Probe sets were mapped to U133 plus 2.0 Array and filtered as described in *Additional methods*. A total of 2,718 unique genes were retained and subjected to R package "Weighted Gene Co-expression Network Analysis (WGCNA)” to identify co-expressed gene modules. A). Optimization and selection of power for adjacency transition of gene-gene correlation matrix (power =7). B). Cluster dendrogram of the gene co-expression modules represented by different colors. Seven gene co-expression modules were detected by hierarchical clustering using dynamic tree cut algorithm integrated in WGCNA with the following parameters: power=7, minModuleSize=120, mergeCutHeight= 0.3. Unclustered genes (genes not correlated with other genes) were collected in Grey module.

**Additional Figure S2. Gene interaction network of IPF prognostic predictor genes.** Significant gene interaction networks were determined using Ingenuity Pathway Analysis (IPA) software. Node shapes denoting different functions were depicted in right panel box. Green and red denote down and up-regulated genes, respectively.

**Additional Figure S3.** **Concordance of IPF prognostic predictor genes between training and each validation cohort.** The fold change of each gene between predicted low-risk and high-risk prognosis patients was plotted between training (X-axis) and validation cohort (Y-axis).

**Additional Figure 4S. Receiver-Operating-Characteristic (ROC) analysis of genomic model for diagnosis prediction.** ROC curves of UCV cohort consisting of IPF patients and healthy individuals were plotted based on the Prognostic Index (PI) derived from IPF genomic model. AUC (Area-Under-Curve) is displayed in the graph. The red line denotes 10% false alarm (1-Specificity).
