# Supplementary figures and images for: A functional genomic model for predicting prognosis in idiopathic pulmonary fibrosis
Source: BMC Pulm Med. 2015 Nov 21;15:147. doi: 10.1186/s12890-015-0142-8 (PMC4654815; doi:10.1186/s12890-015-0142-8)

## Slide 1
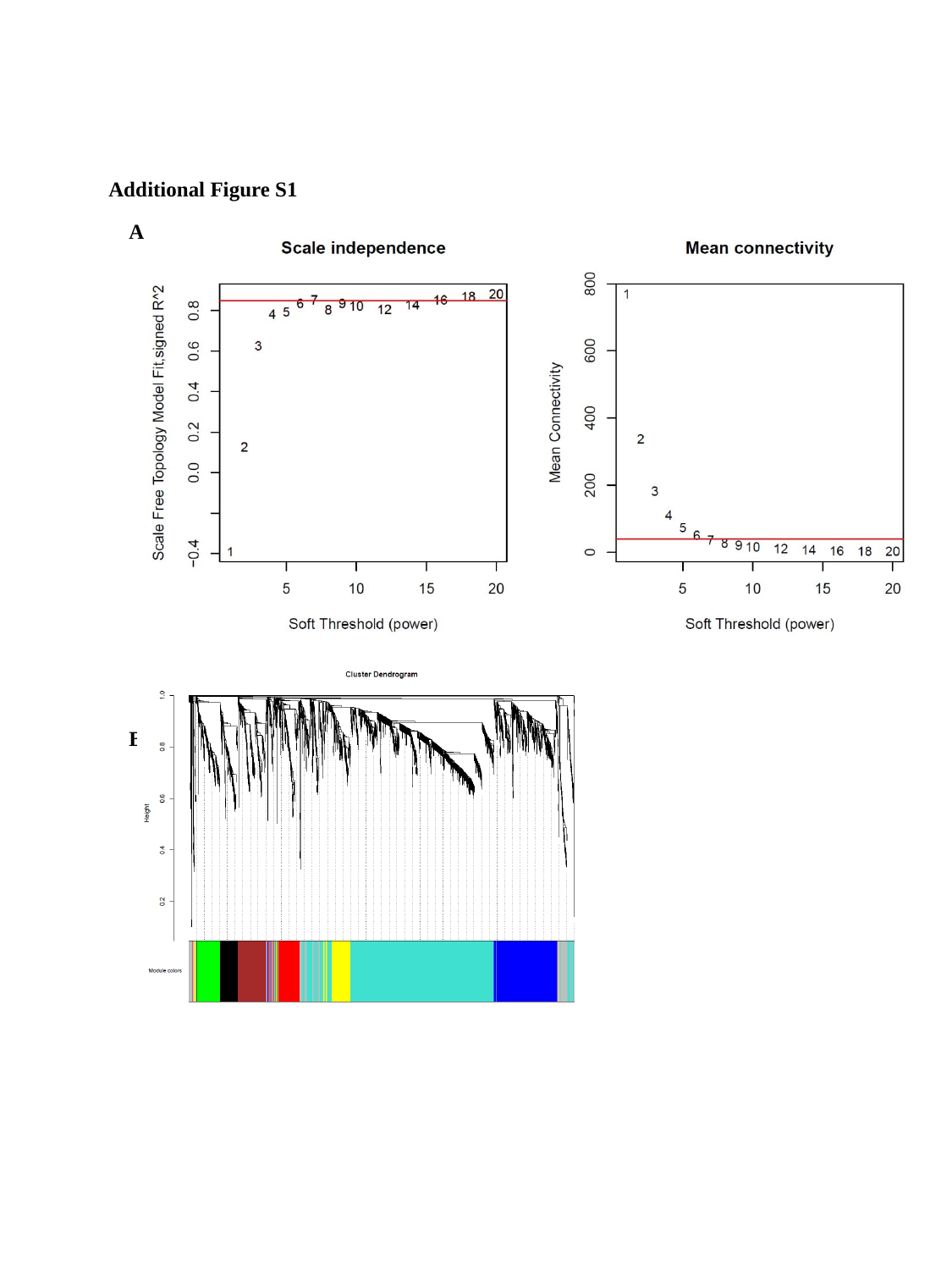

Additional Figure S1
A
B

Supplement: Additional file 2: Figure S1. — Detection of gene co-expression modules in training cohort. Gene expression intensities obtained from Exon 1.0 ST Array were normalized. Probe sets were mapped to U133 plus 2.0 Array and filtered as described in Additional file 1. A total of 2,718 unique genes were retained and subjected to R package “Weighted Gene Co-expression Network Analysis (WGCNA)” to identify co-expressed gene modules. A). Optimization and selection of power for adjacency transition of gene-gene correlation matrix (power =7). B). Cluster dendrogram of the gene co-expression modules represented by different colors. Seven gene co-expression modules were detected by hierarchical clustering using dynamic tree cut algorithm integrated in WGCNA with the following parameters: power=7, minModuleSize=120, mergeCutHeight= 0.3. Unclustered genes (genes not correlated with other genes) were collected in Grey module. (PPTX 187 kb) [file 12890_2015_142_MOESM2_ESM.pptx]

## Slide 1
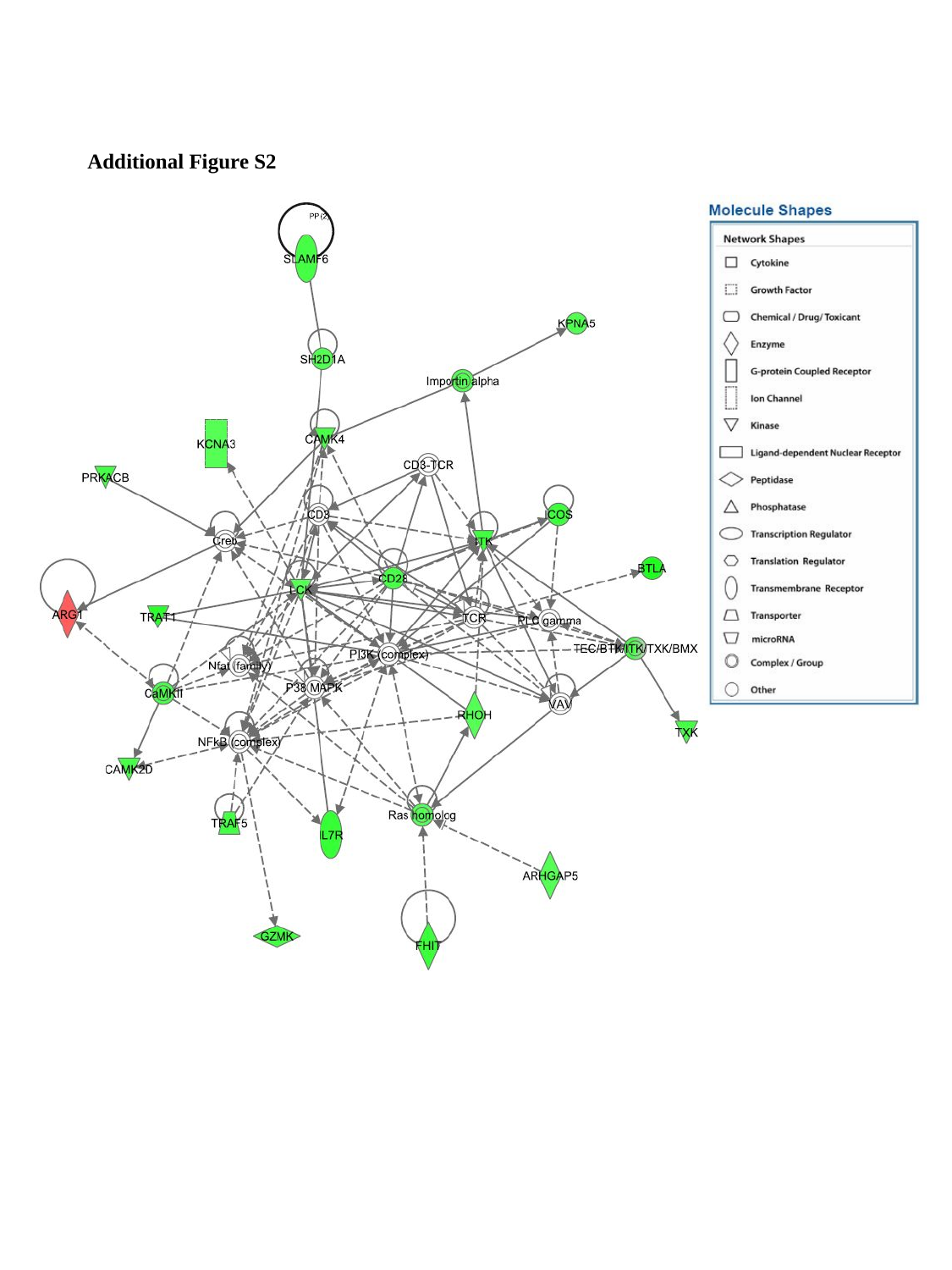

Additional Figure S2

Supplement: Additional file 3: Figure S2. — Gene interaction network of IPF prognostic predictor genes. Significant gene interaction networks were determined using Ingenuity Pathway Analysis (IPA) software. Node shapes denoting different functions were depicted in right panel box. Green and red denote down and up-regulated genes, respectively. (PPTX 544 kb) [file 12890_2015_142_MOESM3_ESM.pptx]

## Slide 1
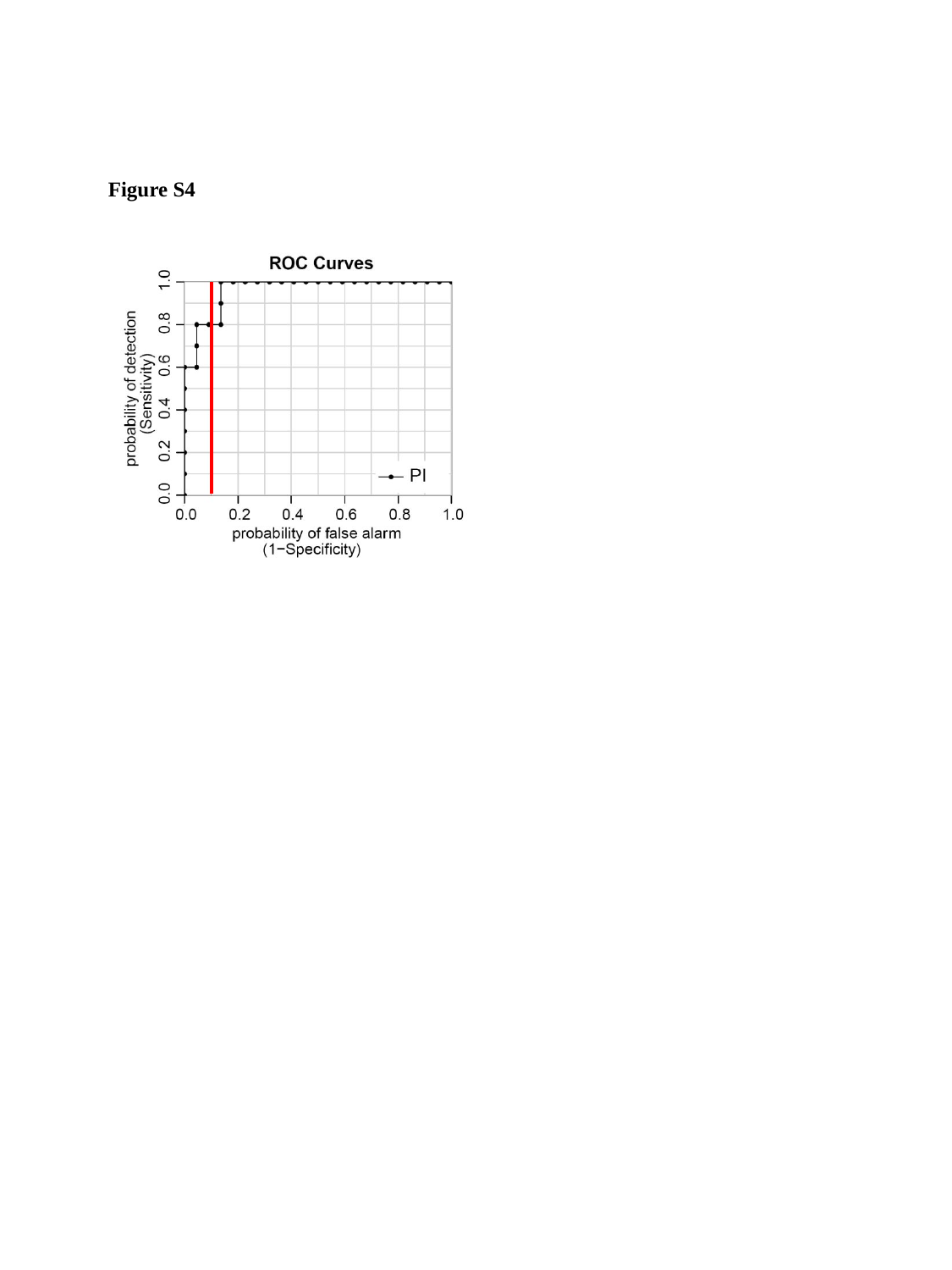

Figure S4

Supplement: Additional file 5: Figure S4. — Receiver-Operating-Characteristic (ROC) analysis of genomic model for diagnosis prediction. ROC curves of UCV cohort consisting of IPF patients and healthy individuals were plotted based on the Prognostic Index (PI) derived from IPF genomic model. AUC (Area-Under-Curve) is displayed in the graph. The red line denotes 10 % false alarm (1-Specificity). (PPTX 79 kb) [file 12890_2015_142_MOESM5_ESM.pptx]
