# Supplementary material for: A functional genomic model for predicting prognosis in idiopathic pulmonary fibrosis
Source: BMC Pulm Med. 2015 Nov 21;15:147. doi: 10.1186/s12890-015-0142-8 (PMC4654815; doi:10.1186/s12890-015-0142-8)
Supplement: Additional file 4: Figure S3. — Concordance of IPF prognostic predictor genes between training and each validation cohort. The fold change of each gene between predicted low-risk and high-risk prognosis patients was plotted between training (X-axis) and validation cohort (Y-axis). (PPTX 51 kb) [file 12890_2015_142_MOESM4_ESM.pptx]

## Slide 1
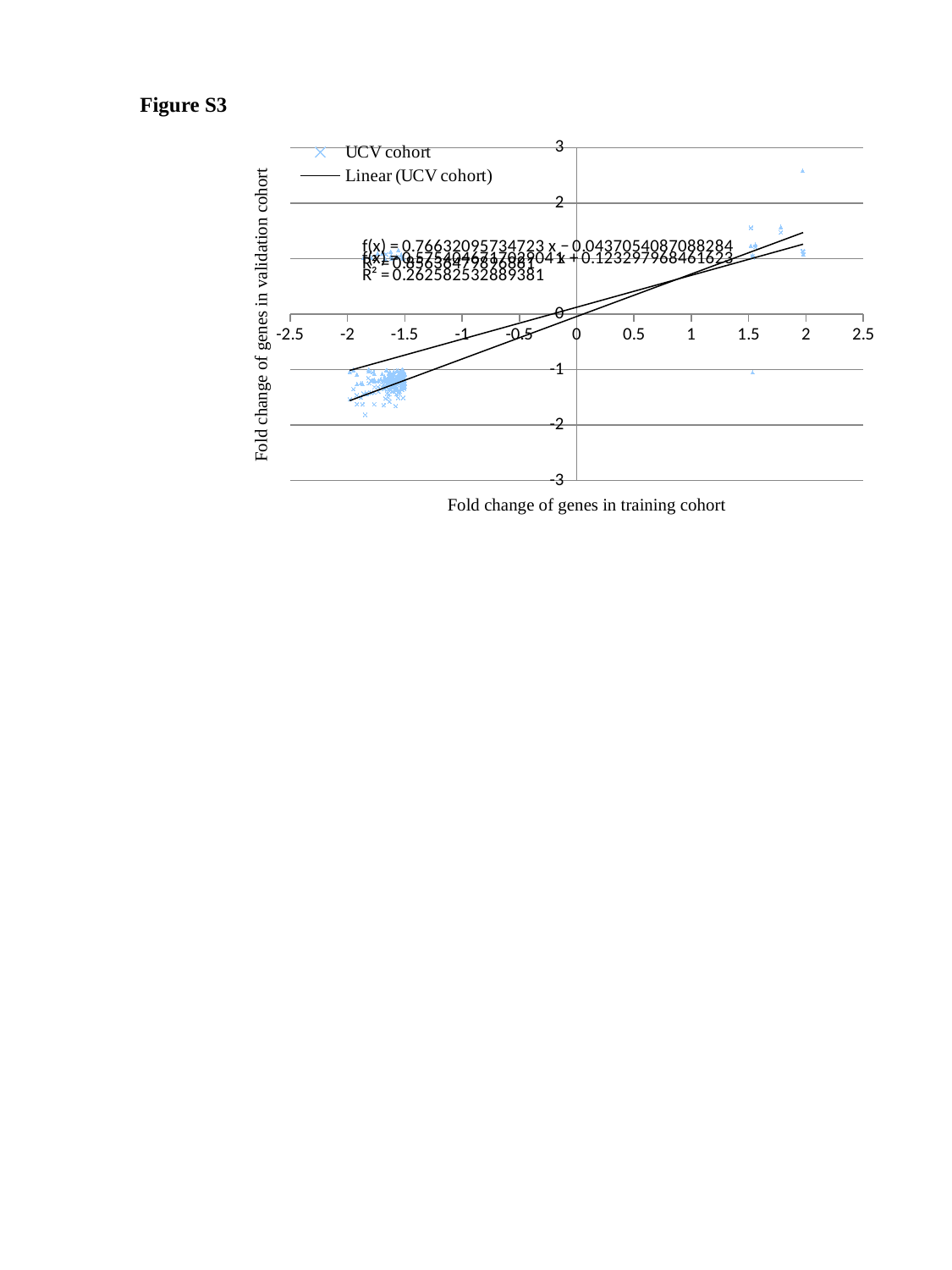

Figure S3
### Chart
| Category | | |
|---|---|---|Fold change of genes in validation cohort
Fold change of genes in training cohort
